# OP43 Co-Creation Of An Evidence-Based Tool To Assess Health Applications To Manage Depression (EvalDepApps Project)

**DOI:** 10.1017/S0266462325101013

**Published:** 2025-12-29

**Authors:** Carme Carrion, Aïna Fuster-Casanovas, Sònia Moretó Melero, Noemí Robles, Andrea Duarte-Díaz, Daniela Cabutto, Josep Vidal-Alaball, Estel Estel Gelabert, Corpus Gómez-calderón, Janaina Ferreira Cavalcanti, Meritxell Davins-Riu, Antoni Pérez-Navarro

## Abstract

**Introduction:**

The use of apps represents a revolution in mental health because they are fast, versatile, manageable, available twenty-four seven, empower patients and professionals, and can reduce stigmatization. However, there is not yet a standardized method to assess effectiveness and safety. The objective of the EvalDepApps project is to develop an evidence-based tool to evaluate apps whose main aim is to manage depression.

**Methods:**

The EvalDepApps project followed several stages: (i) identification of health apps for depression through systematic mapping of the marketplaces; (ii) analysis of effectiveness of mobile health interventions for treating depression through a meta-analysis; (iii) identification by healthcare professionals and users (n=30) of a set of criteria to specifically evaluate apps for depression through a two-round modified Delphi study; and (iv) identification of requirements to be implemented in the EvalDepApps tool through co-creation workshops (design thinking methodology) in three settings (17 healthcare professionals and 13 patients). Currently, the app is being piloted by 15 healthcare professionals and 15 patients.

**Results:**

Thirty apps were identified in marketplaces. Twenty-nine randomized controlled trials were included in the meta-analysis. The analysis showed the most common elements in digital health interventions, the significant effect of mobile health interventions in reducing symptoms (95% confidence interval [CI]: −0.87, −0.37; I^2^=87%), and that hybrid interventions (mobile health plus face-to-face sessions) were the most effective. The Delphi study was based on 51 criteria and reached consensus in 28 criteria; co-creation workshops identified elements of interest for end users (ranking of the apps and recommendation systems). All these elements have been implemented in the EvalDepApps tool, which is being piloted currently.

**Conclusions:**

Mobile health interventions can be effective in reducing depressive symptoms. It is important to standardize evaluation tools to identify which are the most effective. A human-centered approach improves app engagement and effectiveness. Thanks to inputs given by professionals and patients, together with existing evidence, EvalDepApps will be a tool to assess health apps based on a robust methodological approach.

